# A scalable machine-learning approach to recognize chemical names within large text databases

**DOI:** 10.1186/1471-2105-7-S2-S3

**Published:** 2006-09-26

**Authors:** Jonathan D Wren

**Affiliations:** 1 Advanced Center for Genome Technology, Department of Botany and Microbiology, The University of Oklahoma, Norman, Oklahoma 73019, USA

## Abstract

**Motivation:**

The use or study of chemical compounds permeates almost every scientific field and in each of them, the amount of textual information is growing rapidly. There is a need to accurately identify chemical names within text for a number of informatics efforts such as database curation, report summarization, tagging of named entities and keywords, or the development/curation of reference databases.

**Results:**

A first-order Markov Model (MM) was evaluated for its ability to distinguish chemical names from words, yielding ~93% recall in recognizing chemical terms and ~99% precision in rejecting non-chemical terms on smaller test sets. However, because total false-positive events increase with the number of words analyzed, the scalability of name recognition was measured by processing 13.1 million MEDLINE records. The method yielded precision ranges from 54.7% to 100%, depending upon the cutoff score used, averaging 82.7% for approximately 1.05 million putative chemical terms extracted. Extracted chemical terms were analyzed to estimate the number of spelling variants per term, which correlated with the total number of times the chemical name appeared in MEDLINE. This variability in term construction was found to affect both information retrieval and term mapping when using PubMed and Ovid.

## Introduction

Automated approaches to term identification within large corpora are becoming increasingly valuable as the amount of text-based information increases. MEDLINE, for example, is growing at a rate of ~4% per year, with approximately 672,000 new records added in 2005. Manual identification of key terms within large corpora such as this is tedious, time-consuming, costly, difficult to evaluate in terms of error rates, and often involves a minimum amount of expertise. Thus, data-mining approaches to identify trends, associations and patterns within text are being used more frequently [1,2] and an increased emphasis has been placed upon this automated recognition and extraction of terms within text [3], which usually serves as a stepping-stone towards other informatics applications, such as creating term association networks. These networks can in turn be used to identify commonalities shared by a set of terms [4,5] or to infer new associations between individual terms [6]. Accurate term recognition is also essential to automated or semi-automated database creation [7,8] and for straightforward information retrieval.

Considerable effort so far has centered upon the recognition of gene and protein names [9,10], presumably because of the growing number of high-throughput technologies in genomics, transcriptomics and proteomics. Surprisingly, not as much attention has been paid to the recognition of chemical names within text. Not only do most fields utilize chemistry, even aside of obvious ones such as pharmacology, toxicology, biochemistry and genetics, but chemical metabolites are an integral part of the proteomic and transcriptional network that has been the focal point for efforts thus far. Because new chemicals are constantly being synthesized, reference databases become increasingly out-of-date from the time they are first compiled. There is also a persistent need for computer aided indexing of relevant terms within text and a means of extracting chemical information from published sources faster and cheaper. For example, the American Chemical Society and PubChem have both spent considerable time and effort constructing their chemical databases, and the issue of who should pay for access has recently become a topic of controversy [11].

Spelling variation has long been recognized as a problem in information retrieval [12], and chemical names are known to be problematic. Early approaches to matching chemical names utilized approximate string matching techniques [13], such as the use of n-grams to compare chemical names [14]. Most approaches developed to handle these spelling variations, however, are designed with the intention of matching a user-entered term with one or a few entries within a database of chemical names. Identifying or "tagging" chemical names within text is another matter – authors are usually concerned more with reporting their results and less concerned with adhering to nomenclature standards, especially when it is clear to others in their field what they are referring to.

Heuristic approaches have been employed to identify chemical names within text, searching for characteristic patterns found in chemical names such as core chemical prefixes or suffixes, with one study reporting 93% precision and 86% recall [15]. However, only 55 abstracts were used in the evaluation, and they were obtained by searching on keywords related to acetylation, thus it is not at all clear how extensible these results are to larger corpora or other chemical names. Wilbur compared segmentation and Naïve Bayesian (NB) classification methods of recognizing chemical names, training and testing on the UMLS Metathesaurus [16]. However, the segmentation approach is again based upon component term definitions and it is not clear how adaptable it is to new morphological constructions of chemical names. The NB method yielded an impressive 96% precision, but it was tested using the same dictionary it was constructed from, and it is not clear how well it would perform on new terms, especially those buried within scientific text, which contains less standardized terms than are found within a curated database.

## Using an MM to Identify Chemical Names

We hypothesized that a first-order Markov Model (MM) could be used to effectively discern chemical names, due to the fact that chemical component terms (also known as "morphemes") have a large fraction of consistent patterns that might make them highly amenable to MM analysis. After being trained to recognize both chemical and non-chemical names, an MM should be able to effectively judge new words by how well they conform to these patterns. A machine-learning approach such as a MM offers several advantages over heuristic approaches: First, it is capable of learning by example and is more tolerant of ambiguity. Second, MMs conveniently offer statistical confidence scores associated with term classification, and permit a user to adjust precision and recall rates to suit their needs by merely changing the confidence score cutoff. Third, by adjusting the training set, an MM-based approach can attempt to distinguish specific types of chemical compounds (e.g. sulfamides, phosphodiesterases) if the user is interested in such. Finally, no specialized lexicon or dictionary of prefixes/suffixes is required for term recognition. The trade-off, however, is that a large training set is required.

We anticipate that this machine-learning approach will perform much better in identifying chemical formulas than trade or brand names (e.g. Sulforhodamine-101 is better known as "Texas Red"). Trade names are designed specifically to sound more like English words than chemical names, making them easier to pronounce and shorter so they may be remembered more easily. Arguably, this is the biggest drawback of using this method. However, such trade names are often defined proximal to the chemical name within text. Thus, accurately locating the chemical name provides a way to potentially identify and associate both names. The ChemID database contains a mixture of the two.

## Systems and Methods

Code was developed on a Pentium IV 3 GHz machine with 1 GB RDRAM and 109 GB of SCSI hard drive space, using Visual Basic 6.0 (SP5) with ODBC connections to an SQL database. The National Library of Medicine graciously provided XML files containing MEDLINE records with publication dates ranging from 1966 to March 2004 (which includes records indexed prior to publication), as well as a copy of the ChemID plus database , which contained 367,821 records at the time of this study. Only chemical entries in English were processed (some chemicals have foreign name equivalents).

Originally, to train the MM to recognize non-chemical words, we tried to obtain abstracts we thought would not have many chemical names, by drawing from fields we viewed as substantially different from chemistry, such as astronomy, optics, anthropology, math and demography. Upon testing, however, we found many chemical names therein. The optimal training set, of course, would be a random sample of the corpus to be scanned minus chemical names. Since this is not available and each of these "non-chemical" abstract sets was found to have chemical names, we decided to go with an older scientific corpus that would not contain most or any modern chemical names. For this, we used Charles Darwin's "Descent of Man" and "Origin of Species" combined (3.1 MB of text total). For testing, we used Tolstoy's novel "War and Peace" (3.2 MB of text). The electronic full-text of all these corpora can be obtained via Project Gutenberg .

A first-order Markov Model (MM) was developed based upon 2 character transition frequencies. The MM is termed "first-order" because it is assumed that the next state is dependent only upon the current state. Higher-order MMs can take into account more previous states. Words within text are treated as consecutive strings of ASCII symbols, which will be primarily alphanumeric but may also contain non-alphanumeric symbols. A state transition matrix was constructed to track transitions between characters with ASCII values ranging from 32 to 125. Because characters outside this ASCII range occur at generally low frequencies in both words and chemical names, they are all treated as "wildcard" characters having a value of zero. Also, brackets ("][") are converted to parentheses prior to MM evaluation, but are preserved when entering putative chemical names found within text into a database. All words were formatted prior to MM scoring by removing surrounding parentheses, brackets and quotation marks (single & double). Furthermore, certain "tag-along" word prefixes and suffixes were noted where the rightmost hyphen effectively ended the chemical name and began the next English word (e.g. "methoxy-specific", "non-hydrochloride", "glutamine-rich"). Words were stripped of suffixes that followed the following patterns prior to processing: ('t, 's, (s), -or, -to, -and, -the, -old, -rich, -specific). The prefix "non-" was also stripped. These represented the more common patterns and are not an exhaustive list of "tag-alongs". Only chemical names 255 characters in length or shorter were recorded. After processing all MEDLINE abstracts, only 65 chemical names were found to be > 255 characters long. Long chemical names are anticipated to be more common in full-text analysis, since abstract space is usually restricted.

**Figure 1:**
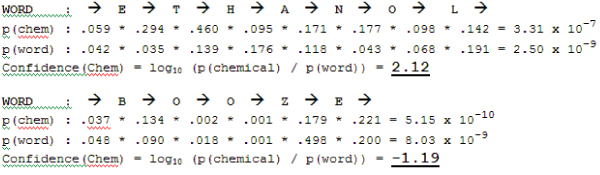
Example of the principle by which a first-order MM works using the words "ethanol" and "booze". State transition frequencies are calculated for each letter in a word (including spaces on both sides of the word) and compared with models the MM has been trained on; in this case chemicals and words. The probability of observing a sequence of letters within each model is calculated as the product of each state (character) transition. To reflect a statistical distance between two models, the log_10 _ratio is taken.

When using an MM to identify and classify a string of characters, the goal is to determine the most likely sequence of tags (states) that generates the characters in the word. In other words, given a word V, calculate the sequence U of tags that maximizes P(V|U). A paper by Rabiner [17] provides a good tutorial on the use of MMs (including HMMs) overall, while Charniak discusses MMs more specifically within the context of sentence and word processing [18]. Because there is a considerable amount of literature on MM theory and construction, it will not be discussed in detail here. Figure 1, however, provides an example of how words are scored and evaluated under different MM models.

To discriminate between the highest scoring model and the next highest, their log_10 _ratio is calculated. This ratio reflects the statistical confidence in choosing the highest scoring model over the next highest and is referred to as the confidence score (which is sometimes also referred to as the log-likelihood ratio). Because some chemical names are very long (>150 characters), probabilities were calculated using a double-precision floating-point variable (8 bytes). To avoid sparseness of the state transition matrix (STM), a pseudo-value of 0.001 was used in place of zero-values. This way, the probability of a given character sequence not previously observed within one model is penalized, but does not automatically assign a value of zero regardless of the other characters observed. Alternatively, one could use a pseudo-value of 1/(# of states)^2, which would be closer to 0.0001, but initially, the MM was case-insensitive and had a lower number of states, so the value was closer to 0.001.

## Results

**Figure 2:**
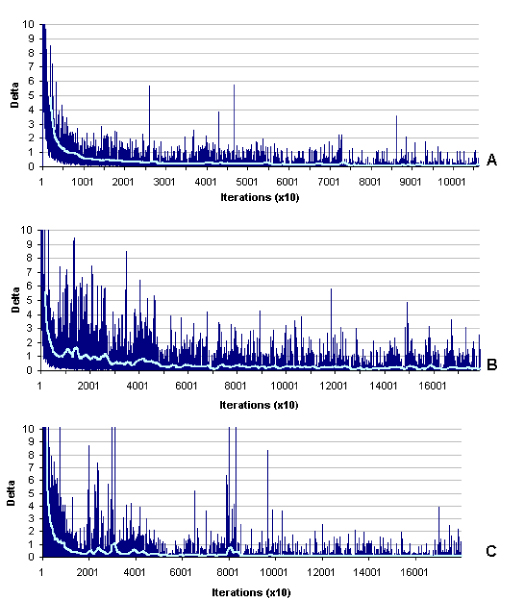
MM training curves converge at different rates (light blue line = 200-period moving average). **(A) **MM training on non-scientific text – in this case, Tolstoy's "War and Peace". Note that convergence is faster and more stable than when trained on scientific text **(B)**, which is more complex. **(C) **Training on chemical names requires a relatively large training set, but reaches convergence.

A first-order MM was first trained using approximately the first half of the ChemID database, which consisted of 194,000 entries (primary names + synonyms). Values of the state transition matrix were tracked during the training to observe model convergence, and gain an idea of how many examples are required to effectively train the MM on chemical names. Figure 2 graphically illustrates the results of the training, where changes in the state transition matrix (STM) values are summed each time the MM is trained on a new word, yielding a value (delta) that corresponds to how much the STM probabilities have changed (given as an absolute value) upon seeing a new example. For example, if the probability of transitioning from the letter A to the letter B was 0.03 after X examples were observed and upon seeing example word X+1, this value changed to 0.02, the magnitude of this change would be 0.01 and delta would be calculated by summing all the changes. In these graphs, delta is calculated over every 10 training examples. As more training examples are used, fewer new patterns are seen by the MM and delta should converge towards zero. Without the character handling mentioned in the Systems and Methods section, a stable convergence was difficult to reach even with 194,000 examples. However, when rare characters are treated as "wildcards" (e.g., relatively rare characters such as "~" and "|" would be treated as the same symbol), MM convergence occurs faster (data not shown). For comparison, training set convergence is shown for words in scientific abstracts (Figure 2b), words closer to the spoken language (Figure 2a), and chemical names (Figure 2c).

## MM Recall Rates on ChemID Test Set

Abstracts are processed by sentence and each sentence is broken into words by separating the sentence based upon the presence of spaces. Only words with at least 4 characters are scored. Because chemical names can either be contiguous or a series of terms (e.g. "chloroquine sulfate", "13-Docosynoic acid", "Glycine ethyl ester hydrochloride"), proximal terms are concatenated when each term is above the scoring threshold.

After MM training, a test set of 20,000 entries was selected from ChemID that were not included in the training set. The entire test set should be correctly predicted to be chemicals, so any that were not so predicted were classified as false-negatives (FNs). Each individual word within the ChemID entry was passed to the MM for evaluation in the same manner words within sentences would be processed (e.g. if the entry was "2-hydroxy-benzyl alcohol" then each word would be sent separately). By definition, there are no false-positives in this set, so this step was designed to estimate recall rates. Precision was measured by testing the MM on a set of non-chemical words and determining how many were falsely classified as chemicals.

**Figure 3:**
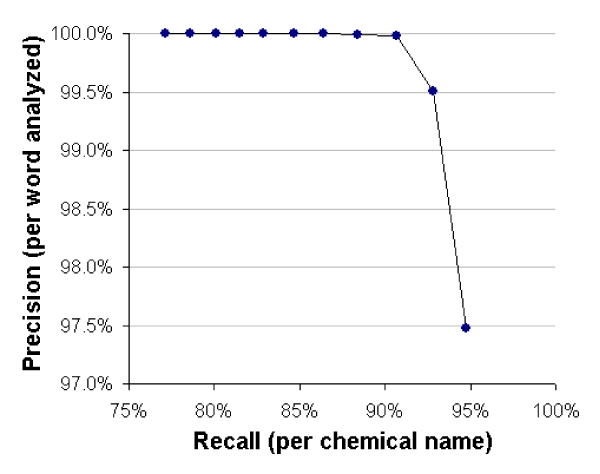
The effects on precision and recall rates from using a cutoff score. Test sets containing chemical names and words were evaluated with an MM trained on both types of data and cutoff scores ranging from 10 to zero were used to define which entries were valid. The data points from left to right reflect the precision and recall rates obtained by using each cutoff value, shown in descending order from 10 (far left) to zero (far right). The optimal tradeoff between precision and recall appears to be somewhere between a cutoff of one and two.

From these 20,000 entries, there were a total of 9,723 unique terms (e.g. the word "methyl" appeared in many different chemical names, but for evaluation purposes was only counted once). Without using a cutoff score, a total of 509 chemical component terms (5%) were erroneously classified as words and 7,697 out of 304,650 non-chemical words submitted (2.5%) were erroneously classified as chemicals. However, there were only a total of 480 unique words within these 7,697. Figure 3 shows how the choice of a cutoff score affects the precision and recall of the system, with recall being defined as True Positives/(True Positives + False Negatives) and precision being defined as True Positives/(True Positives + False Positives). Precision is also commonly referred to as specificity and recall as sensitivity. Most entries receiving very low scores (i.e. those classified as a word with high confidence) did not contain chemical groups, but were more like alphanumeric identifiers (e.g. U-58176, K442), while others were words used to describe a chemical compound or its preparation (e.g. brilliant, brown, mixture, bark, powder).

## Recognizing full chemical names

The previous step measured the ability of the MM to identify individual component terms, but not the ability to recognize entire terms, which will be important when analyzing text. The number of components within a chemical name was also tracked, and the ability of the MM to correctly identify all components of a term was also high. The first 20,000 chemical terms of the test set were processed, totaling 8,283 entries. Of these, 6,243 database entries had all their component terms receive a score above 1 (75.4%), 1,781 terms had at least one component term receive a score above 1 (21.5%) and 258 terms had no component scoring above 1 (3.1%). It was particularly problematic for the MM to correctly identify short terms (1–4 characters) found in some chemical names (e.g. "Actinomycin D", "Strychnos gum" "Antifoam B"), especially if the term was a trade name or description (e.g. "Sun Yellow K", "Swascofix E 45", "Idet 20").

Note that even though precision per word is very high (see Figure 3), this small error rate can translate into many errors when analyzing large corpora. For example, if we assume MEDLINE contains 7 million abstracts at an average 200 words per abstract that equals 1.4 billion words. At a 1% error rate, that would total approximately 14 million errors. Not all errors will necessarily come from different words, and so the total number of unique errors may be far different. To evaluate this, we processed all of MEDLINE, using a cutoff score of 1, which is estimated by these measurements to yield approximately 92.8% recall (per chemical name present) and 99.5% precision (per word analyzed).

## MM Precision on MEDLINE abstracts

A total of 13,154,593 MEDLINE records were processed, containing 7,439,689 abstracts. Each word at least 4 characters long within an abstract was sent to the MM for evaluation using a cutoff score of 1. Each word scored as a chemical name was marked within the abstract and consecutive words receiving a cutoff score greater than one were concatenated into a single entry for deposition into an SQL database. Exceptions were made when a comma followed by a space was found within the entry. In these cases, the entries were treated as separate chemicals (i.e. a list). If a chemical name was found more than once, a frequency counter was incremented for that term.

After all abstracts had been processed, a total of 33,929,499 putative chemical recognition events were recorded, yielding a total of 1,052,654 unique putative chemical names recognized within MEDLINE. From this database, random samples of 100 entries were taken for several ranges of cutoff scores. Each putative chemical name entry in this database was extracted with its surrounding sentence for manual evaluation. Random sampling was repeated 3 times to permit an average and standard deviation to be calculated. A database entry was considered to be correct if the entry was the name of a chemical compound, a chemical group or side-chain, or the name of a family of chemical compounds. If an extra word flanked the chemical on either side, it was not considered an error as long as the rest of the term was a chemical name. For example, in the entry "hydrolyzed alpha-napththyl acetate", the word "hydrolyzed" is not part of the proper name, but the proper name "alpha-napththyl acetate" is nonetheless identified and thus not counted as an error. Similarly, the entry "disoxaril/ml" was extracted from the sentence "0.3 mg disoxaril/ml" and not counted as an error. Protein entries were counted as errors, but not single amino acids. Gene names were also counted as errors, unless the gene was named after a chemical process (e.g. Alkaline Phosphatase). Table 3 summarizes the results of this analysis. The number of false-positive errors rises significantly upon large-scale analysis of a biomedical corpus, but fortunately remains within a reasonable range. The most problematic entries were those with the lowest cutoff, as expected, but it was encouraging to see a significant number of chemical names receiving high scores.

A number of words erroneously recognized as chemicals had at least one syllable that closely resembled a common pattern found within chemical names (e.g. "examine", "suicide", "prophylactic", "paramecium"), or were descriptive words used in chemical nomenclature (e.g. cyclic, solid, hydrophilic, alpha).

## Chemical Name Variance Within MEDLINE

Although the International Union for Pure and Applied Chemistry (IUPAC) has set forth well-established guidelines on chemical nomenclature (see ), and software is also available to generate standard IUPAC names from chemical structures and vice versa , it is not known how consistently authors follow these conventions. Table 1 offers an example of morphological (spelling) variation for the chemical 8-SPT, illustrating the many permutations that can be derived from a single chemical name. To test how this variation affects information retrieval, several of the more common variations of this chemical name were selected. First, Ovid was used to map the terms to a medical subject heading (MeSH term). Second, the PubMed search engine was queried using the same terms to see how many articles would be retrieved. Table 2 shows that this variation in the construction of a chemical name is not necessarily a trivial matter, since it affects the number of records retrieved per PubMed query as well as attempts at mapping terms to subject headings. In the cases where PubMed found over 200 entries, the term was successfully mapped to the MeSH term "8-(4-sulfophenyl)theophylline", which is classified as a "substance name". This dependency upon specific term construction profoundly affects information retrieval. Thus, we sought to get a better estimate of how much variation there is within MEDLINE in the construction of chemical names.

Our goal was to identify chemical names that are used to refer to the same chemical compound, yet vary in their morphological construction. Table 1 offers a good example of the considerations that must be taken into account while doing this. For the chemical 8-SPT, the most common morphological construction is 8-(p-sulfophenyl)theophylline. Within the list, a number of variations on this can be seen. Existence and placement of either parentheses or hyphens account for most of the variation, but there is also a variation within component terms. The "p" is used to indicate the presence of a para group, and in five out of 24 cases is not present. Some entries, apparently errors, list this "p" as a "4" or a "rho", both of which strongly resemble a "p" (perhaps errors that occurred during data-entry or optical character recognition?). In 8 out of 24 cases, the sulfur group is spelled with a "ph" instead of "f". Finally, one variant spells "theophylline" without the "e", which is probably a spelling error rather than an intentional variation. These last few examples suggest that simple rules for comparing terms, such as removing punctuation and spaces, will not adequately capture term variation. Aligning terms and evaluating them by percent similarity would solve these problems, but raises another. In some cases, such variation is critical to the nature of the compound. For example, consider enantiomer designations of L-alanine versus D-alanine, which can also be represented as (+)-alanine or (-)-alanine, amines versus amides or imines, methyl groups versus ethyl groups, R versus S chirality, and numeric placement of side chains. All these terms vary by one character, and thus evaluating them by percent similarity would falsely suggest that these types of terms are equivalent when they are not. An imperfect solution is proposed so that an estimate can be obtained, by using a heuristic acronym-definition resolution routine to resolve acronyms when explicitly defined within the text [19,20]. The rationale and assumption for pairing chemical names with their acronyms is that authors should usually be aware of which minor (few-character) distinctions signify a closely related yet chemically distinct compound and will strive to reflect this distinction when defining an acronym for the compound, to avoid both nomenclature ambiguity and confusing the reader. We first test how well this assumption holds up by examining how many of the R/S, D/L and (+)/(-) variants in chemical names are both defined using only one acronym.

## Pairing Chemical Names with their Acronyms

Querying the January 2004 version of the ARGH database, 435 acronym-definition pairs were found beginning with either "(+)" or "(-)", and 9 acronyms were used for both the (+) and (-) designations; for the 1,514 acronym-definition pairs beginning with the patterns "R-*" or "(R)*" or the patterns "S-*" or "(S)*", 8 acronyms were used for both the R and S designations; and for the 3,380 acronym-definition pairs beginning with either D- or L-, 22 acronyms were used for both the D and L designations. On average then, within the samples taken, a unique acronym does not distinguish stereo descriptor variants 0.7% (39/5329) of the time. From this, it seems reasonable to assume that pairing chemical names with their acronyms permits an additional quality control check when trying to ensure that the variation observed between two similar terms is indeed variation of the same chemical name. A disadvantage is that not all chemical names will have their acronyms defined within each abstract, but this should not prevent obtaining an estimate of term variance.

Definitions from the ARGH database were scored by the MM and their acronym retrieved if they were scored as a chemical name. Each definition having the same acronym as the chemical name was then aligned using a dynamic programming algorithm similar to the FASTA algorithm[21]. A similar approach using BLAST was successful in identifying gene names within text [22], although in this case we are adding an additional control to ensure the two terms refer to the same compound. If two definitions shared the same acronym as well as 75% of their characters upon alignment, they were considered spelling variants. Analysis was restricted to chemical names with a confidence score of 1 or greater and those that were mentioned within MEDLINE at least twice. All comparisons were conducted in a case-insensitive manner. A total of 12,155 acronym definitions were classified as chemical names, and 4,345 had at least one spelling variant. A total of 15,288 variants were detected by these criteria, giving an average number of spelling variants per chemical name of 1.26. However, because the distribution in the number of variants found follows an inverse power-law distribution, taking a simple average is perhaps not the most appropriate means of expressing variation – there is an extreme disparity in how many times each of these chemical names is mentioned within MEDLINE. A more informative way of visualizing this variation is to examine the relationship between the number of times a chemical name is published and the number of spelling variants observed.

**Figure 4:**
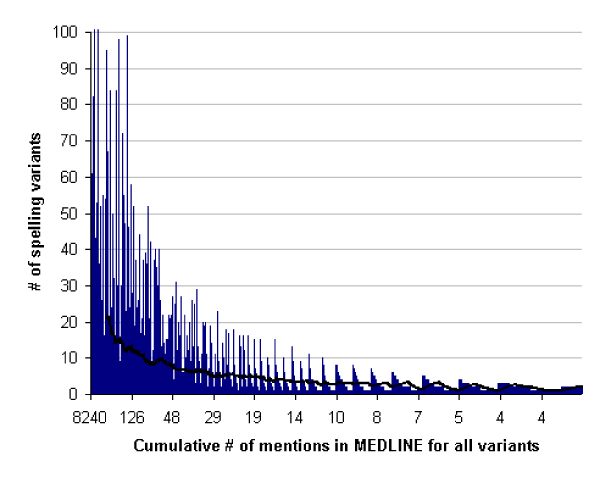
The number of spelling variants for a given chemical name is correlated with the number of times it has appeared within the literature. Here the number of spelling variants per chemical name is plotted against the total number of times the chemical name and all variants appeared within MEDLINE. The black line represents a 100-period moving average.

As Figure 4 shows, the number of variants per chemical name was correlated with the number of times the chemical name appeared in the literature, suggesting that variation in the precise construction of many chemical names is, to a degree, inevitable and a function of how many times it is written. Some names, such as norepinephrine, are mentioned many times within MEDLINE yet have very little variance because they have few component terms. Using the 510 chemical names that had their acronyms defined at least 50 times within MEDLINE, whether a spelling variant was detected for them or not, we found that 490 (96%) had at least 1 variant. When the most commonly observed spelling form is expressed as a percentage of the total number of observations for chemical names (see Table 1 for an example), on average it comprises the majority of total definitions observed (71% ± 26%). This suggests that the most common spelling form of any chemical name can be used to retrieve the majority of occurrences of that chemical name within a textual database, but will fail to retrieve a large percentage of significant records in the absence of methods to identify potential variants.

## Discussion

The results of this report suggest that using a Markov Model provides a straightforward and accurate way to recognize chemical names within any textual source. The overall precision rates were highly encouraging; especially considering such a large corpus had been analyzed. The biggest limitations of the MM in identifying chemical names within text were two-fold: First, and most noteworthy, there were many "tag-along" prefixes and suffixes that accompanied many of the chemical names. As a consequence, the total number of unique chemical names in MEDLINE would be overestimated by this study (e.g. "doxorubicin" and "doxorubicin-based" should be the same, but have separate entries). Second, and particularly for the lower-scoring chemical names, the MM was unable to correctly determine starting and ending points for the named chemical, especially when one of the ends is defined by a short (1–4 character) word. Encouragingly, though, because the MM does have a relatively high precision in identifying chemical morphemes, that provides a good starting point for further refinements in extracting the full-name. This did not seem to be as much of a problem as when chemical prefixes were separated by English words – the MM was able to pick up the prefixes, but not to pair them with their name (e.g. "Activities for tyrosine-phenol were measured along with the related substitutions – toluene and -benzene").

One possibility for further refinement that was not explored in this report is the construction of a higher-order MM. That is, a MM that takes into account transition frequencies between 3 or more characters at once. As per the example given in Figure 1, this would entail calculating the probability for both words and chemicals that the letters e-t-h would occur in that order for each model. Because the STM would become sparser (i.e. there would be more transition values equal to zero because certain transition patterns will never be observed within a given model and limited training set), it is reasonable to hypothesize that a gain in precision could be achieved. However, it is neither clear how recall would be affected, nor the size of the required training set. Also a possibility for improvement, specifically in the area of identifying the shorter prefixes, suffixes and infixes associated with chemical names, is the construction of a Hidden Markov Model whereby word substructures are represented as the hidden state and the model trained to maximize the probability of a phrase boundary being reached.

## Figures and Tables

**Table 1:** Example of morphological (spelling) variations for a chemical, 8-SPT, as observed within MEDLINE abstracts. 8-SPT is also found abbreviated as 8SPT, 8SPTH, and 8-PSPT.

**#**	**Chemical Name**	**# times observed**	**% of total**
1	8-(p-sulfophenyl)theophylline	13	19.1%
2	8-sulfophenyltheophylline	10	14.7%
3	8-sulphophenyltheophylline	8	11.8%
4	8-(p-sulphophenyl)theophylline	5	7.4%
5	8-(p-sulfophenyl)-theophylline	4	5.9%
6	8-(p-sulphophenyl)-theophylline	4	5.9%
7	8-(p-sulphophenyl) theophylline	3	4.4%
8	8-(p-sulfophenyl) theophylline	3	4.4%
9	8-p-sulpho-phenyltheophylline	2	2.9%
10	8(p-sulfophenyl)theophylline	2	2.9%
11	8-(sulfophenyl)theophylline	1	1.5%
12	8-p-sulfophenyl theophylline	1	1.5%
13	8-(4-sulfophenyl)theophyline	1	1.5%
14	8 (p-sulphophenyl) theophylline	1	1.5%
15	8-(sulfophenyl) theophylline	1	1.5%
16	8-p-sulfophenyltheophylline	1	1.5%
17	8-p-sulphophenyltheophylline	1	1.5%
18	8(p-sulfophenyl)-theophylline	1	1.5%
19	8-rho-(sulfophenyl)theophylline	1	1.5%
20	8-sulphophenyl-theophylline	1	1.5%
21	8-(p-sulfophenyl)-theophyllin	1	1.5%
22	8-(para-sulfophenyl)theophylline	1	1.5%
23	8-p-sulfophenyl-theophylline	1	1.5%
24	8-(p-sulfophenyl)-theophylline)	1	1.5%

**Table 2:** 8-SPT as an example of how variation in chemical nomenclature affects information retrieval. Here, Ovid was used to map 5 variations of the chemical name 8-SPT to subject headings. Only adenosine and theophylline were common to each variant tested. 8-SPT is an adenosine receptor antagonist. For comparison, PubMed retrieved 341 unique records when using each of the keywords separated by "OR" in the query.

Chemical Name mapped to term(s)	8-p-sulphophenyltheophylline	8-sulphophenyltheophylline	8-p-sulfophenyltheophylline	8-SPT	8SPT
**Adenosine**	X	X	X	X	X
Adenosine Triphosphate		X		X	X
Aorta		X			
Autonomic Nervous System	X				
Cerebellum			X		
Cerebral Cortex	X				
Coronary Circulation			X		
Coronary Vessels				X	
Creatine Kinase					X
Endothelium, Vascular		X			
Heart				X	
Hippocampus					X
Hyperhomocysteinemia				X	
Iris	X				
Muscle Contraction	X				
Muscle, Smooth			X		
Myocardial Reperfusion Injury					X
Neurons					X
Neutrophils			X		
Parasympathetic Nervous System	X				
Phenethylamines		X			
Rats, Wistar				X	
Receptors, Adrenergic, alpha-1	X				
Receptors, Cell Surface			X		
Receptors, Purinergic	X		X	X	
Receptors, Purinergic P1		X	X	X	
Receptors, Purinergic P2		X			
Spinal Cord		X			
Synaptic Transmission	X				
**Theophylline**	X	X	X	X	X
Vasodilation		X		X	
Xanthines			X		

**# of documents retrieved via PubMed**	**22**	**251**	**47**	**297**	**15**

**Table 3:** Scalability of MM term evaluation for chemical names when applied to a large corpus, in this case approximately 13.1 million MEDLINE records that contain approximately 7.4 million abstracts. Using these estimates, the overall precision for chemical term entry into the database is 82.7%.

**Cutoff**	**Sample 1 FP**	**Sample 2 FP**	**Sample 3 FP**	**Avg. Precision**	**Stdev**	**# Records**	**Errors (est.)**
**1–2**	42%	46%	48%	54.7%	3.1%	203,985	92,473
**2–5**	27%	25%	22%	75.3%	2.5%	319,000	78,687
**5–10**	5%	3%	5%	95.7%	1.2%	202,655	8,782
**10–20**	2%	0%	1%	99.0%	1.0%	164,286	1,643
**21+**	0%	0%	0%	100.0%	0.0%	162,728	-
	**Weighted Average**			82.7%	**Total**	**1,052,654**	**181,584**
